# Network analysis identifies a gene biomarker panel for sepsis-induced acute respiratory distress syndrome

**DOI:** 10.1186/s12920-023-01595-8

**Published:** 2023-07-13

**Authors:** Duan Zhu, Mi Zhou, Houli Zhang, Liang Gong, Jianlin Hu, Hu Luo, Xiangdong Zhou

**Affiliations:** 1 Department of Respiratory and Critical Care Medicine, the First Affiliated Hospital of Army Medical University (Southwest Hospital), No.30 Gaotanyan Main Street, Chongqing, 400038 China; 2 Department of Biochemistry and Molecular Biology, Army Medical University, Chongqing, China

**Keywords:** Acute respiratory distress syndrome, Sepsis, Network analysis, Gene biomarker panel, AUC

## Abstract

**Background:**

Acute respiratory distress syndrome (ARDS) is characterized by non-cardiogenic pulmonary edema caused by inflammation, which can lead to serious respiratory complications. Due to the high mortality of ARDS caused by sepsis, biological markers that enable early diagnosis are urgently needed for clinical treatment.

**Methods:**

In the present study, we used the public microarray data of whole blood from patients with sepsis-induced ARDS, patients with sepsis-alone and healthy controls to perform an integrated analysis based on differential expressed genes (DEGs) and co-expression network to identify the key genes and pathways related to the development of sepsis into ARDS that may be key targets for diagnosis and treatment.

**Results:**

Compared with controls, we identified 180 DEGs in the sepsis-alone group and 152 DEGs in the sepsis-induced ARDS group. About 70% of these genes were unique to the two groups. Functional analysis of DEGs showed that neutrophil-mediated inflammation and mitochondrial dysfunction are the main features of ARDS induced by sepsis. Gene network analysis identified key modules and screened out key regulatory genes related to ARDS. The key genes and their upstream regulators comprised a gene panel, including EOMES, LTF, CSF1R, HLA-DRA, IRF8 and MPEG1. Compared with the healthy controls, the panel had an area under the curve (AUC) of 0.900 and 0.914 for sepsis-alone group and sepsis-induced ARDS group, respectively. The AUC was 0.746 between the sepsis-alone group and sepsis-induced ARDS group. Moreover, the panel of another independent blood transcriptional expression profile dataset showed the AUC was 0.769 in diagnosing sepsis-alone group and sepsis-induced ARDS group.

**Conclusions:**

Taken together, our method contributes to the diagnosis of sepsis and sepsis-induced ARDS. The biological pathway involved in this gene biomarker panel may also be a critical target in combating ARDS caused by sepsis.

**Supplementary Information:**

The online version contains supplementary material available at 10.1186/s12920-023-01595-8.

## Introduction

Acute respiratory distress syndrome (ARDS) is a type of respiratory failure characterized by rapid and widespread inflammation of lungs, accompanied by hypoxemia, reduced lung compliance, and chest imaging examination showing bilateral alveolar opacity [1]. Globally, there are more than 3 million ARDS patients each year, and it accounts for 10% of the patients admitted to intensive care units (ICU) [2]. While, the overall prognosis of ARDS is poor, with a mortality of approximately 40% [3]. Furthermore, the survivors are usually accompanied with adverse sequelae, such as exercise limitation, physical and cognitive impairment [4].

Sepsis is the most common trigger of ARDS and the highest cause of ARDS mortality [5]. Clinical research has shown that ARDS related to sepsis has a worse recovery a higher overall disease severity and a higher mortality rate than non-sepsis-related ARDS [6]. As we know, sepsis is the body’s extreme response to an infection. Once the immune response of the body to infection is dysregulated, resulting in the inability to clear the infection, sepsis will develop through pro-inflammatory immune mechanisms. The latest definition from the NIH NHLBI panel states that sepsis is a severe endothelial dysfunction caused by both intravascular and extravascular infections, resulting in damage to the microcirculation [7]. The severe inflammatory response caused by sepsis can lead to changes in the permeability of lung epithelial cells and capillary endothelial cells. The influx and apoptosis of alveolar macrophages and neutrophils eventually lead to diffuse alveolar injury and severe hypoxia, which are the clinical features of ARDS [8]. In addition, the clinical study of Michelle Ng Gong, et al. showed that pneumonia-induced severe sepsis is more likely to develop ARDS than those with extrapulmonary sources of infections [9]. However, ARDS is a highly heterogeneous syndrome. The plasma molecular alterations of ARDS resulted from various causes are different [10]. And not all sepsis patients develop ARDS. The current treatment of ARDS is not significantly different from that of patients with sepsis, of which mechanical ventilation remains the preferred life-saving strategy. It cannot identify or predict the progression of ARDS in patients with sepsis, and cannot reduce the mortality of patients [11]. Therefore, the development of early diagnostic biomarkers and a specific treatment for sepsis-induced ARDS are essential.

High throughput gene analysis was a powerful tool to reveal the key pathways and genes of diseases. In recent years, it has also been applied to the research of ARDS. A Genome-wide association studies pointed out several candidate genes were related to the development of ARDS, including the interleukin 6 (IL6), interleukin 10 (IL10), interleukin 1 receptor antagonist (IL1RN), vascular endothelial growth factor A (VEGFA; also known as VEGF), angiotensin-converting enzyme (ACE), soluble mannose-binding lectin 2 (MBL2) and visfatin (NAMPT) [12]. Acosta-Herrera et al. found the correlation between VEGF signaling, neuron projection morphogenesis and ARDS by using the lung tissue of animal model of sepsis [13]. Wang et al. compared polymorphonuclear neutrophil (PMN) transcriptome alterations in sepsis patients and ARDS patients, and proposed that GAPDH, MAPK8, PIK3CB and MMP9 may play an important roles in the progression of ARDS [14]. These results not only helped us to further understand the mechanism of sepsis induced ARDS, but also proved that the analysis of potential ARDS related genes and pathways based on gene expression characteristics may be a breakthrough to further understand the genetic mechanism of ARDS.

Recently, with the development of analytical techniques in systems biology, gene network analysis has been widely used in disease-related high-throughput omics studies [15]. Gene network analysis can catalog, integrate and quantify the molecular interactions at the genomic scale, and identify key network features associated with disease processes, which provided an excellent complement to the traditional single-gene approach to research [16, 17].

**Fig. 1:**
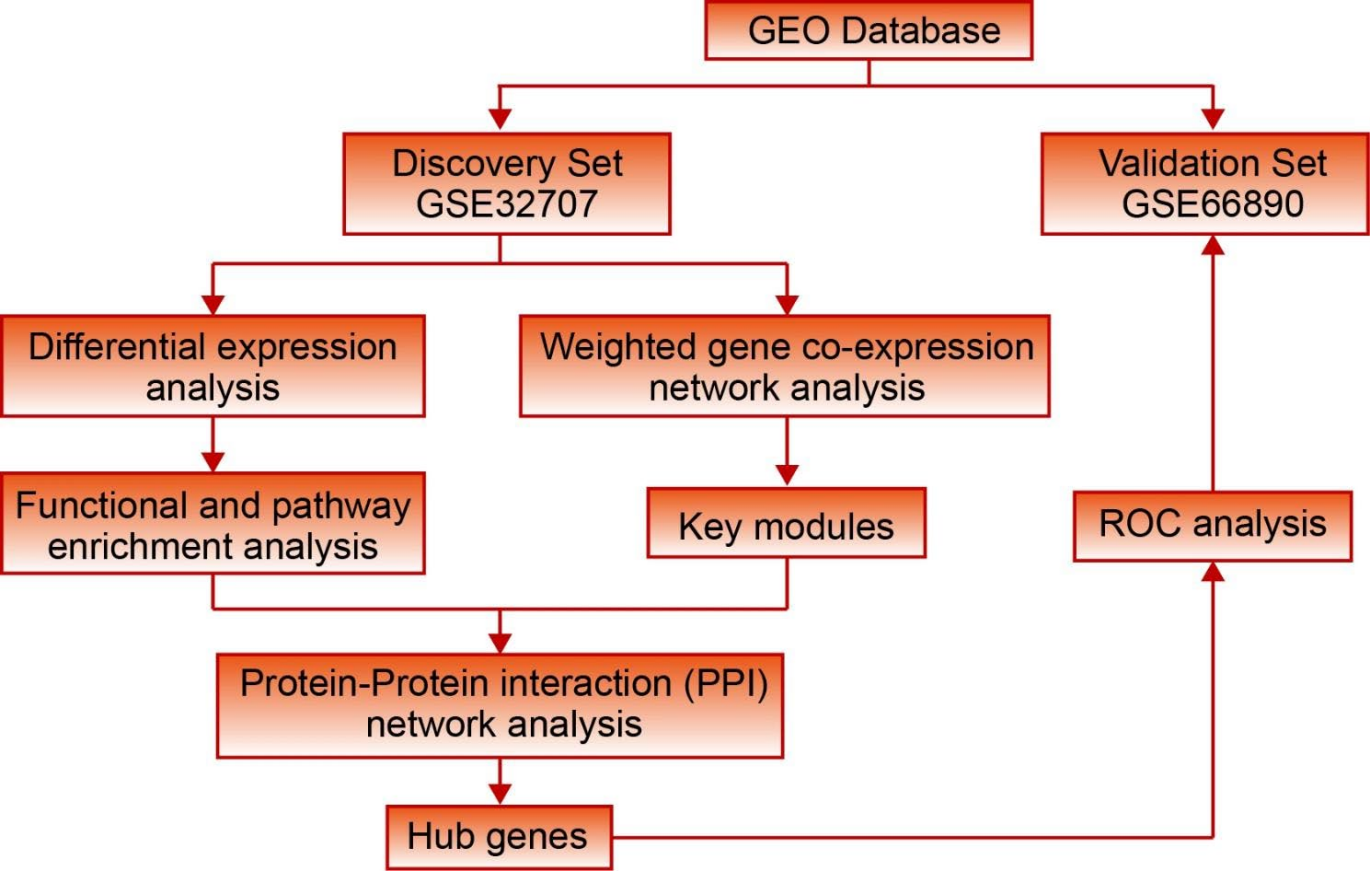
Workflow of this study. A discovery dataset and a validation dataset were downloaded from the GEO database. The integrated analysis of gene expression and gene network was performed on the discovery dataset to identify hub genes related to sepsis-induced ARDS. The identified hub genes were performed ROC analysis in the validation set to demonstrate their efficacy

In this study, we conducted an integrated analysis on expression level and network level of the whole blood microarray profiles of pneumonia-induced sepsis patients, sepsis-induced ARDS patients and healthy controls (Fig. 1). The network approach was adopted to identify the key genes and biological processes closely related to the development of ARDS and predict the possible upstream regulatory factors. Our results showed the panel composed of these genes was a potential biomarker of sepsis induced ARDS, which may be helpful to better understand the occurrence and development of ARDS.

## Materials and methods

### Microarray data acquisition

The Gene Expression Omnibus (GEO) database (https://www.ncbi.nlm.nih.gov/geo/) is a free global public database storing genomics and transcriptomics data, including high-throughput sequencing and microarray expression files. We searched the GEO database for ARDS-related studies and found two datasets that met our requirements, including samples of healthy controls, pneumonia-induced sepsis alone, and ARDS developed from pneumonia-induced sepsis. The larger sample size dataset (GSE32707) was used as the discovery set, and another dataset (GSE66890) was used as the validation set. Institutional Review Board approval was not required, because our study was based on a public database and did not involve in animal or human samples.

Dataset GSE32707 was submitted by Dolinay et al. and approved by the Partners Human Research Committee [18]. The dataset contains 123 whole blood samples, including 58 patients with sepsis alone, 31 patients with sepsis-induced ARDS, and 34 healthy controls (Table S1). The detailed diagnostic criteria and demographic information can be found in the original manuscript [18].

The validation dataset GSE66890 was submitted by Kangelaris et al. and approved by the University of California, San Francisco Institutional Review Board [19]. The dataset included 28 patients with sepsis alone and 29 sepsis patients with ARDS (Table S1). The normalized data was available and downloaded directly. The information of patients, the collection of blood samples and the process of the generation of microarray profile were described in detail in the published manuscript [15].

### Data pre-processing

The raw data was corrected background and quantile normalization using limma package of R (ver 4.0.3) [20]. Outlier samples were detected by calculating standardized sample network connectivity Z-scores, and samples with Z-score < -2.5 were removed [21]. Then we clustered samples using hclust tool and removed samples with the farthest distance from other samples. BiomaRt package was used to transformed Illumina probes to gene symbols. Only protein-coding genes were kept in our study. CollapseRows function was used to combine multiple probes annotated to the same gene symbol.

### Differential expression analysis

In the present study, we used limma package of R to identify differential expressed genes (DEGs) between sepsis-alone group, sepsis-induced ARDS group and healthy control group [22]. Benjamini-Hochberg (BH) method was used to estimate false discovery rate (FDR) [23]. Adjusted *p*-value less than 0.05 was used as the threshold of significance.

### Functional and pathway enrichment analysis

Gene Ontology (GO) annotation and enrichment analyses were performed using Gene Set Enrichment Analysis (GSEA) and Ingenuity Pathway Analysis software (IPA, http://www.ingenuity.com). These two approaches represent two different philosophies on the alteration of gene function. GSEA allows the use of all genes to investigate the alterations of biological functions caused by disease, enabling us to observe which biological functions tend to be up-regulated and which to be down-regulated [24]. In contrast, the canonical pathway analysis used by IPA prefers to know which pathways are primarily involved in DEGs, and thus the same pathway may contain both up- and down-regulated genes, given that these genes may have activated or repressive interactions with each other. We used both methods and potentially got complementary results that provide more accurate information. We used WebGestaltR package of R to perform GSEA based on no redundant GO biological process databases (1,000 permutations). The result was considered as significant which absolute value of normalized enrichment score (|NES|) more than 1.5 and FDR less than 0.25 [25]. Subsequently, we used IPA to implement over-represent analysis of canonical pathways for DEGs of multiple comparisons. One-sided fisher’s exact *p*-values were calculated to filter significance (*p* < 0.05).

### Weighted gene co-expression network analysis (WGCNA)

Gene co-expression network is a widely used approach to explore the correlation relationship structure of gene cooperative alterations in disease status. In this study, we used WGCNA package of R to identify co-expression clusters based on all genes [26, 27]. Briefly, we calculated the gene correlation matrix and converted it to an adjacency matrix. Next, a signed weighted correlation network was constructed based on a fit to scale-free topology. Dynamic tree cut method was used to detect co-expressed gene clusters, called modules. The detailed parameters were used as follows: networkType = “signed”, corFnc = “cor”, TOMType = “signed”, TOMDenom = “mean”, mergeCutHeight = 0.25, deepSplit = 4, minModuleSize = 30. Each module was labeled by an independent color, and the genes labeled by gray did not belong to any co-expressed gene modules. We identified the key modules related to ARDS based on module-group correlation, the gene-group correlation within the module, and the degree of enrichment of DEGs to the modules. The correlation of module-group was calculated based on module eigengene (ME), which was the first principal component of the module representing the expression of the modules. The module enrichment of DEGs was performed by one-sided fisher’s exact test, and the FDR less than 0.05 adjusted by BH method was used as the threshold of significance. Functional analysis for the key module associated with ARDS was conducted by IPA software based on canonical pathway database.

### Protein-protein interaction (PPI) network analysis and ROC analysis

STRING (https://www.string.org), a web-based database was used to construct the protein-protein interaction network for genes of key modules. The Cytoscape software (ver 3.8.0, https://www.cytoscape.org) was employed for visualizing the PPI network. The CytoHubba plugin (ver 0.1) provided degrees of each node in the PPI network and the top 10 genes were considered as hub genes [28]. Another plugin iRegulon (ver 1.3) was applied to predict the potential upstream regulating factors (URFs) of hub genes, such as transcription factors (TFs) [29]. We analyzed the correlation between the URFs with the highest NES-score and hub genes to establish a regulatory gene panel with significant correlation structure. Logistic regression and receiver operating curve (ROC) analysis were performed to obtain the diagnostic value of this gene panel for sepsis or sepsis-induced ARDS.

## Results

### Data processing of microarray

According to the data pre-processing process described above, we removed 19 outlier samples (Fig. S1). The 47,220 Illumina probes detected in the raw data were annotated to 18,066 protein-coding genes for our subsequent analysis.

### Identification of DEGs

**Fig. 2:**
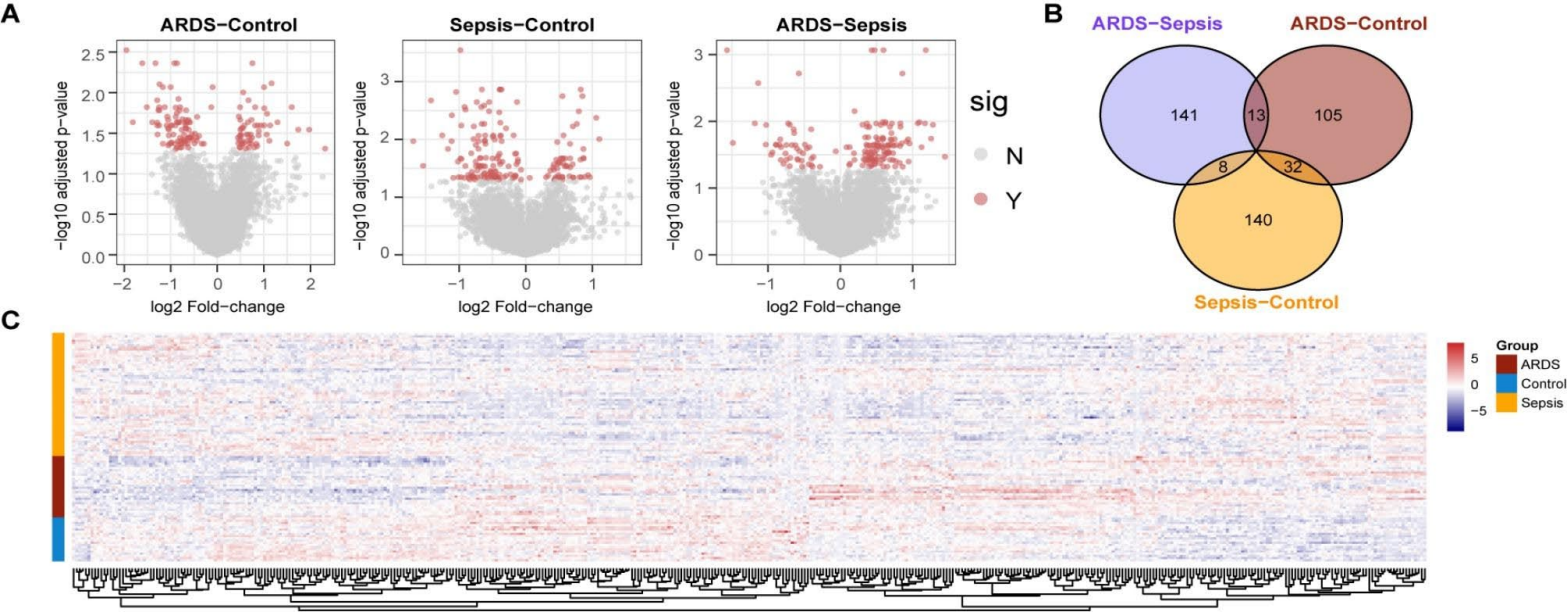
Differential expressed genes analysis (DEGs). (**A**) Volcano plots displays the DEGs in three comparisons. Red points represent DEGs, and gray points represent no significance. (**B**) Venn plots showed the shared DEGs and unique DEGs of multiple comparisons. (**C**) Heatmap showed the change of expression in sepsis-alone group, sepsis-induced ARDS group and control group. Red represents up-regulation and blue represents down-regulation

In total, we identified 439 DEGs (BH-adjusted *p* < 0.05, Fig. 2A, Table S2). Among them, 180 DEGs were identified between sepsis-alone group and control group, 150 DEGs were identified between sepsis-induced ARDS group and control group, and 162 genes were differential expressed between sepsis-alone group and sepsis-induced ARDS group (Fig. 2B). Although ARDS was developed from sepsis, there were few DEGs shared with the sepsis-alone group (nearly 70% of their DEGs were unique). The unsupervised hierarchical cluster heatmap displayed the expression changes of these genes in three groups (Fig. 2C). These results suggested that the expression levels of many genes were disrupted during the development of sepsis into ARDS. It also prompted us that there were some key molecules can serve as the biomarkers for prediction, prevention and treatment.

### Functional and pathway enrichment analysis for DEGs

**Fig. 3:**
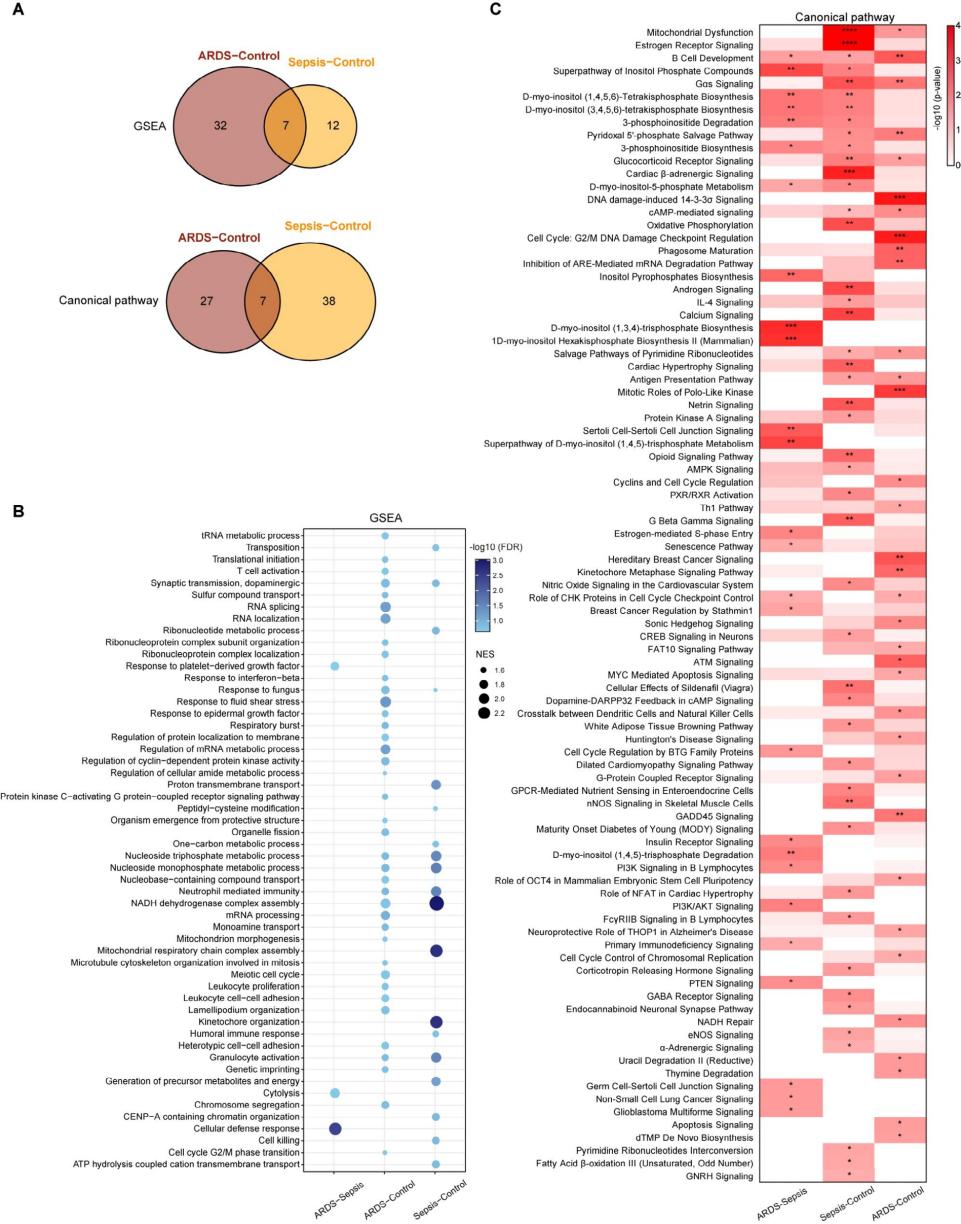
Function and pathway enrichment analysis. (**A**) Venn plots shows the overlap of GO functions and pathways that are significantly enriched in ARDS group and sepsis-alone group. (**B**) The bubble chart shows the result of gene set enrichment analysis (GSEA). The size of points represents the absolute value of normalized enrichment score (|NES|) and the color intensity of each point represents the significance. (**C**) Heat map exhibits the canonical pathways that significantly enriched by DEGs. The color intensity of each grid was scaled by -log_10_(*p-*value). The asterisk is used to indicate significance. *p < 0.05, **p < 0.01, ***p < 0.001, ****p < 0.0001

The enrichment analyses of biological functions and pathways were performed using GSEA and IPA software. Consistent with the result of DEGs, both sepsis-alone group and sepsis-induced ARDS group has independent changes of biological functions or pathway compared with controls (Fig. 3A). A total of 54 GO terms and 90 canonical pathways were significantly enriched based on GSEA and IPA software, respectively (Table S2). We observed that the sepsis-alone group and the ARDS group shared 7 enriched GO functions and pathways, and showed concordant regulatory direction, such as neutrophil mediated immunity, NADH dehydrogenase complex assembly and dopaminergic related pathways (Fig. 3A, Fig S2). The unique dysfunctions of sepsis-alone group mainly included mitochondrial energy metabolism processes, such as oxidative phosphorylation, fatty acid metabolism and some neural pathways. The unique altered functions of ARDS group were mainly involved in cell cycle and apoptosis related functions (Fig. 3B-C).

### Gene co-expression network analysis

**Fig. 4:**
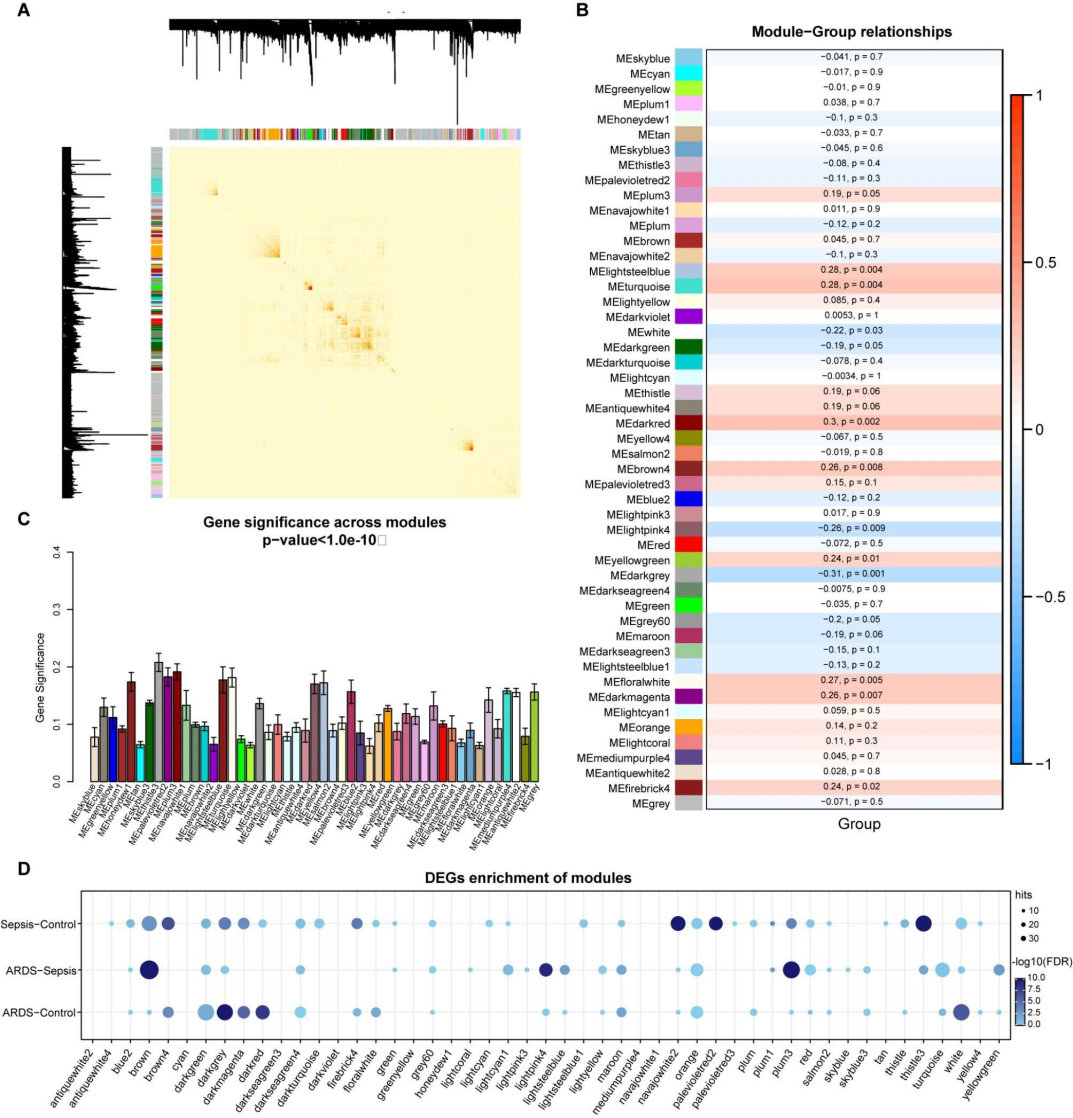
Gene co-expression network analysis. (**A**) Heatmap plot of gene network. The heatmap depicts the topological overlap matrix (TOM) among all genes. Light color intensity represents low overlap and progressively darker red color represents higher overlap. Block of darker colors along the diagonal are the modules. (**B**) Heatmap quantifies module-group associations. Rows are labeled by names and colors of modules. The text of each row indicates the correlation coefficients and *p*-values of the correlation analysis between each module eigengene (ME) and groups. Red means positive correlation and blue means negative correlation. (**C**) Average significance of genes in each module. (**D**) The bubble plot shows the modules which are significantly enriched by DEGs. One-sided fisher’s exact test was performed, and the Benjamini-Hochberg method was used to adjust FDR. The size of each point represents the number of DEGs in each module and the color intensity represents the significance

**Fig. 5:**
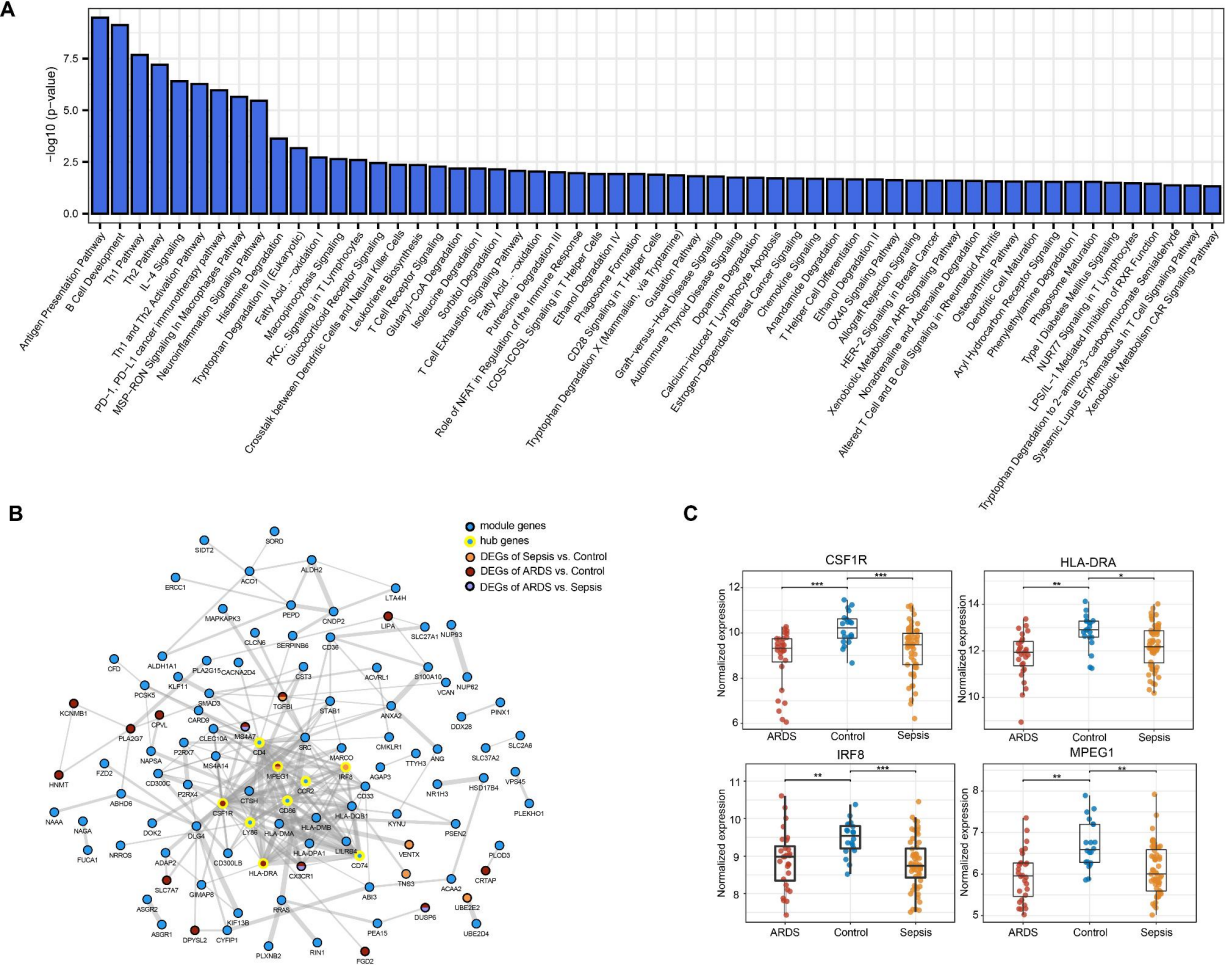
Function analysis and protein-protein interaction (PPI) network of darkgrey module. (**A**) Significantly enriched canonical pathways for darkgrey module. (**B**) PPI network of genes in darkgrey module. (**C**) Boxplots showed the expression of hub genes in three groups

Subsequently, we constructed a signed gene co-expression network for all genes to explore the changes of gene correlation relationships related to ARDS. The detailed parameters were described in Method section. The power of 20 was used to make the network up to scale free fit (Fig. S3A). A total of 49 co-expressed gene modules were detected based on the power estimation of 20 and the size of modules was range from 37 to 1,375 (Fig. 4A, Table S3). In order to identify the modules related to ARDS, we calculated the module-group relationships based on pearson correlation analysis and obtained 13 significant modules (Fig. 4B). Then, we calculated the gene-group relationships in each module (Fig. 4C). The darkgrey module owed the largest correlation coefficient absolute value and module gene significance, thus it was considered as the key module. (Table S3, Fig. 4D). In the functional analysis of the darkgrey module, we found 57 significantly enriched pathways, including numbers of immune/inflammation-related signaling pathways, such as Antigen Presentation Pathway, B Cell Development, Th1/Th2 Pathway, IL-4 Signaling, as well as neuroinflammatory signaling pathways, and fatty acid metabolism pathways (Fig. 5A, Table S3).

### PPI network and ROC analysis

**Fig. 6:**
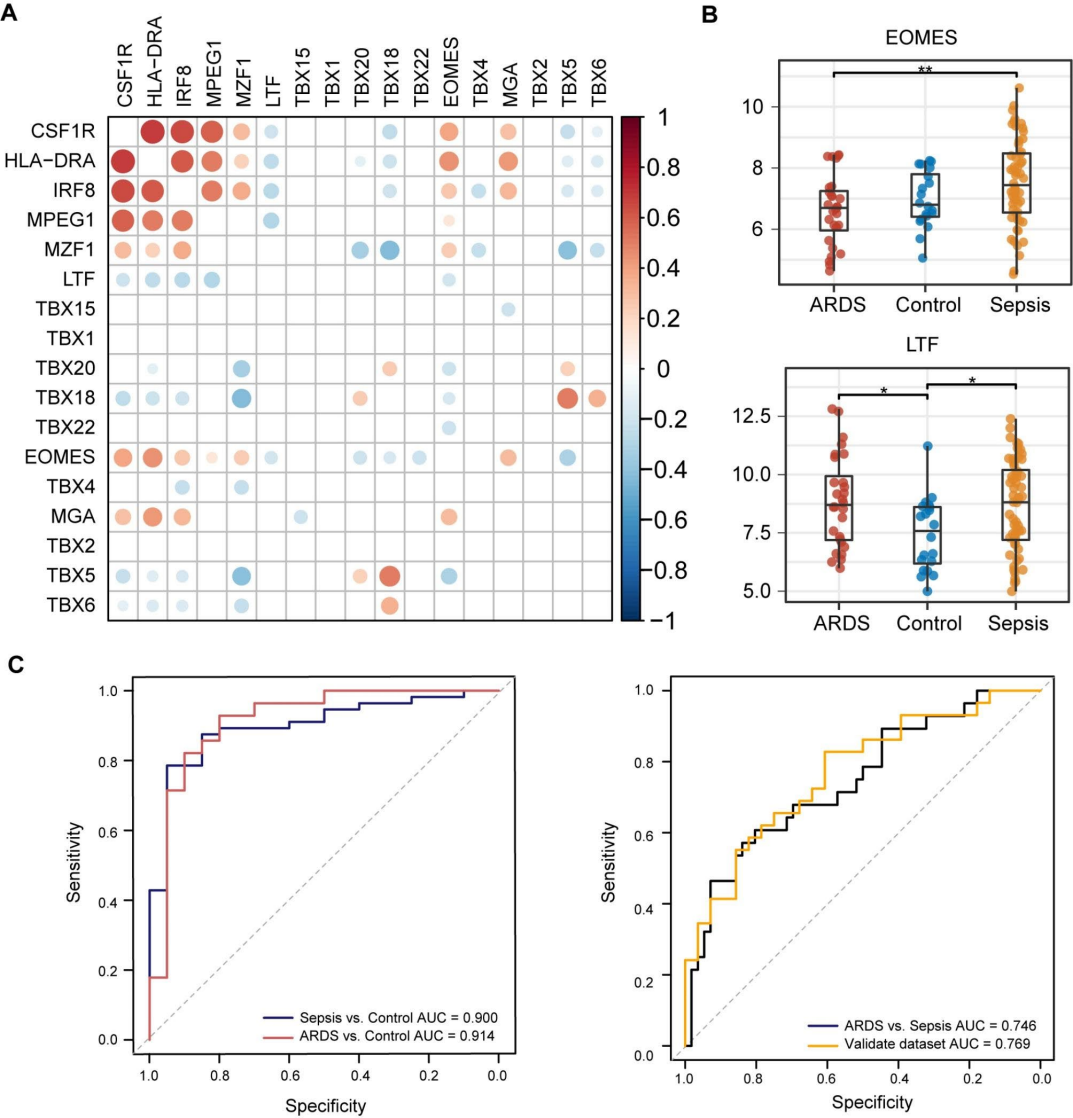
URFs and ROC analysis. (**A**) Correlation analysis between URFs and hub genes. Pearson correlation, *p* < 0.05. (**B**) Expression of URFs. *p < 0.05, **p < 0.01. (**C**) ROC curve analysis of hub genes and URFs. Left panel showed the ROC analysis between disease group and control group. Right panel showed the ROC analysis between sepsis patients and ARDS patients

We constructed PPI network based on the 171 genes in darkgrey module by STRING database (Fig. 5B). The degree of each node was calculated, and the top 10 genes were considered as hub genes (Fig. 5B, Table S4). Four genes of them were differential expressed among sepsis-alone group, sepsis-induced ARDS group and controls, which were CSF1R, HLA-DRA, IRF8 and MPEG1. The plugin iRegulon identified 14 potential URFs upstream of these four key genes based on the largest NES (Table S4), of which 13 URFs were detected by the microarray profiling. MZF1, EOMES and MGA showed significant positive correlation with the hub genes, as well as LTF, TBX18, TBX5 and TBX6 showed significant negative correlation with hub genes (Fig. 6A). Among these potential URFs, EOMES (*p*_*ARDS−Sepsis*_=0.002) and LTF (*p*_*Sepsis−Control*_=0.018, *p*_*ARDS−Control*_=0.013) showed a trend of expression differences among groups (Fig. 6B, Fig. S4). Therefore, we took EOMES, LTF, CSF1R, HLA-DRA, IRF8 and MPEG1 as a united diagnostic panel and assessed their diagnostic efficiency by ROC analysis (Fig. 6C). United gene panel had excellent diagnostic ability in both ARDS and sepsis (AUC_ARDS_=0.914, AUC_Sepsis_=0.900), and can well diagnose ARDS developed by sepsis (AUC_ARDS−Sepsis_=0.746). An independent dataset confirmed (GSE66890) the united gene panel had a potential diagnosis efficiency, with an AUC of 0.769 (Fig. 6C). These results suggested the gene biomarker panel was reliable and robust, which can be used for the diagnosis of sepsis-induced ARDS.

## Discussion

In this study, we performed an integrated analysis of gene expression and gene network levels on microarray expression profiles of sepsis-induced ARDS patients, sepsis patients and healthy controls. In detail, we compared the DEGs between these three groups and explored their functional pathways. Furthermore, we screened the hub genes for sepsis-alone patients and sepsis-induced ARDS patients, and we found that the panel composed of these hub genes featured a good diagnostic efficacy.

Several previous studies have explored risk factors for sepsis-induced ARDS, including pneumonia infection [30], and blood endocan levels [31]. However, few studies have systematically compared sepsis-alone and sepsis-induced ARDS at genetic level. In addition, objective biomarkers for sepsis-induced ARDS are lacking.

In this study, we found that both the biological pathways of GSEA analysis (based on the continuous gene expression features) and IPA analysis (based on the differential gene expression features) suggested that neutrophil-mediated inflammatory response and mitochondrial dysfunction are the major characteristics of ARDS caused by sepsis. As we all know, ARDS was an acute inflammatory disease [8, 32], and neutrophils were considered to be an important component of the inflammatory microenvironment in ARDS [33]. Neutrophils were activated by dual feedback from exogenous and endogenous inflammatory stimuli after lung injury [34]. These activated neutrophils will release cytotoxic substances, such as reactive oxygen species (ROS), telomerase and various pro-inflammatory factors, which will further aggravate the inflammation [35]. In addition, Nguyen et al. and Teixeira et al. showed that neutrophils will promote the development of ARDS by assembling and activating NADH oxidase complexes to produce ROS [36, 37]. This was consistent with our findings that a mitochondrial function-dependent NADH dehydrogenase complex process in ARDS developed from sepsis (Fig. 3B-C). Mitochondria and several ATP-producing genes were the main sources of ROS products, which performed well in predicting the survival rate of ARDS patients [38]. Compared to the sepsis-alone group, we also found that the sepsis-induced ARDS group had some unique dysregulation functions related to cell fate, such as apoptosis signal. Apoptosis of lung endothelial cells (ECs) was one of the main pathological characteristics of ARDS [39]. Several studies have shown elevated levels of ATP or adenosine can promote endothelial cell apoptosis through multiple signaling pathways [40, 41]. Extracellular supplementation of ATP or adenosine can reduce Ras methylation and Ras GTPase activity by inhibiting isoprenylcysteine-O-carboxyl methyltransferase (ICMT), which in turn inhibits the activation of downstream signaling of molecules including Akt, ERK-1 and ERK-2 to induce apoptosis of ECs [40].

Furthermore, co-expression network analysis and PPI network helped us identified four hub DEGs were identified. Among these hub genes, colony stimulating factor 1 receptor (CSF1R), a cytokine which controls the production, differentiation, and function of macrophages, was significantly up-regulated in sepsis-alone group and sepsis-induced ARDS group. Previous evidence showed excessive recruitment and activation of macrophages from the blood, as well as resident alveolar macrophages (AM), may be key factors in the development of ARDS [42–46]. Macrophages can be activated through the classical JAK/STAT1 pathway by binding interferon-γ (IFN-γ) to cell surface receptors [47–49]. The down-regulation of interferon regulatory factor 8 (IRF8) in sepsis patients and ARDS patients promoted inflammatory and infection and activated macrophages through IFN-γ (Fig. 5C) [50]. The decreasing of macrophage-specific marker (MPEG1) and major histocompatibility complex, class II, DR alpha (HLA-DRA) in sepsis patients and ARDS patients were consistent with these findings (Fig. 5C) [51, 52].

Moreover, we also identified two possible upstream URFs of the hub genes, which were significant increased in sepsis patients and ARDS patients (Fig. 5). Lactotransferrin (LTF) has been shown to be a major innate immune responder and played an important role in controlling of the development of acute septic inflammation [53–55]. Although LTF was not a transcription factor, it had a serine protease activity, which can cut arginine-rich regions in a variety of microbial virulence proteins. This function contributed to the regulation of antimicrobial activity [56]. Neutrophils can directly produce LTF, and the release of LTF played a pivotal role in the development and resolution of inflammation [57]. Previous studies showed eomesodermin (EOMES) can promote CD8 T cells producing IFN-γ and their cytotoxicity [58]. In CD4 T cells, EOMES can either induce the production of IFN-γ by Th1 cells or promote Tr1 cells by driving IL-10 production [59, 60]. The regulation of EMOES on T cells and products was consistent with the results of our modular pathway enrichment analysis. Combined with these six hub genes, we found the diagnostic panel was highly efficient in distinguishing the healthy controls, sepsis patients and sepsis-induced ARDS patients.

## Conclusions

In current study, we performed an integrated analysis based on gene expression and gene network and identified key regulators in the development of sepsis to ARDS. A six-gene panel including EOMES, LTF, CSF1R, HLA-DRA, IRF8 and MPEG1 was discovered and validated with a high accuracy both in sepsis subjects and sepsis-induced ARDS subjects. Our findings provide meaningful biomarkers for the diagnosis, and clues for the pathogenic mechanism of sepsis and ARDS.

### Limitations

There were also some limitations in this study. Firstly, this study was limited by the search results of public databases and sample sizes. Secondly, our study was based on bioinformatics methods and screened out the marker genes of sepsis-induced ARDS with high diagnostic efficiency. The diagnostic results of these genes need to be verified in a larger datasets. Thirdly, the lack of in vivo and in vitro proof. For our next work, we will collect a large-scaled clinical samples from multi centers to confirm the stability of the predictive power of these markers, and confirm their therapeutic potential through in vivo and in vitro experiments.

## Electronic supplementary material

Below is the link to the electronic supplementary material.

Supplementary Material 1

Supplementary Material 2

Supplementary Material 3

Supplementary Material 4

Supplementary Material 5

## Data Availability

Publicly available datasets were used for analysis in this study. These data can be obtained from the Gene Expression Omnibus database (GEO, https://www.ncbi.nlm.nih.gov/geo/). The scripts used in this manuscript can be found at https://github.com/xiangdongZhou01/ARDSNetwork.

## Abbreviations

ARDS: Acute Respiratory Distress Syndrome
AUC: Area under the subject working characteristic curve
BH: Benjamini–Hochberg
CSF1R: Colony stimulating factor 1 receptor
DEGs: Differentially expressed genes
EOMES: Eomesodermin
FDR: False discovery rate
GO: Gene ontology
GSEA: Gene set enrichment analysis
HLA-DRA: Major histocompatibility complex, class II, DR alpha
IPA: Ingenuity Pathway Analysis
IRF8: Interferon regulatory factor 8
LTF: Lactotransferrin
MPEG1: Macrophage-specific marker
PPI: Protein-protein interaction
ROC: Receiver operating curve
ROS: Reactive oxygen species
URF: Upstream regulating factor
WGCNA: Weighted gene co-expression network analysis

## References

[1] Force ADT, Ranieri VM, Rubenfeld GD, Thompson BT, Ferguson ND, Caldwell E (2012). Acute respiratory distress syndrome: the Berlin definition. JAMA.

[2] Fan E, Brodie D, Slutsky AS (2018). Acute respiratory distress syndrome: advances in diagnosis and treatment. JAMA.

[3] Lewis SR, Pritchard MW, Thomas CM, Smith AF (2019). Pharmacological agents for adults with acute respiratory distress syndrome. Cochrane Database Syst Rev.

[4] Aeffner F, Bolon B, Davis IC (2015). Mouse models of Acute Respiratory Distress Syndrome: a review of Analytical Approaches, pathologic features, and common measurements. Toxicol Pathol.

[5] Bellani G, Laffey JG, Pham T, Fan E, Brochard L, Esteban A (2016). Epidemiology, patterns of Care, and mortality for patients with Acute Respiratory Distress Syndrome in Intensive Care Units in 50 countries. JAMA.

[6] Sheu CC, Gong MN, Zhai R, Chen F, Bajwa EK, Clardy PF (2010). Clinical characteristics and outcomes of sepsis-related vs non-sepsis-related ARDS. Chest.

[7] Hawiger J, Veach RA, Zienkiewicz J (2015). New paradigms in sepsis: from prevention to protection of failing microcirculation. J Thromb Haemost.

[8] Yang CY, Chen CS, Yiang GT, Cheng YL, Yong SB, Wu MY, et al. New Insights into the Immune Molecular Regulation of the pathogenesis of Acute Respiratory Distress Syndrome. Int J Mol Sci. 2018;19(2). 10.3390/ijms19020588 10.3390/ijms19020588 PMC5855810 29462936

[9] Gong MN, Thompson BT, Williams P, Pothier L, Boyce PD, Christiani DC (2005). Clinical predictors of and mortality in acute respiratory distress syndrome: potential role of red cell transfusion. Crit Care Med.

[10] Calfee CS, Eisner MD, Ware LB, Thompson BT, Parsons PE, Wheeler AP (2007). Trauma-associated lung injury differs clinically and biologically from acute lung injury due to other clinical disorders. Crit Care Med.

[11] Hu Q, Hao C, Tang S. From sepsis to acute respiratory distress syndrome (ARDS): emerging preventive strategies based on molecular and genetic researches. Biosci Rep. 2020;40(5). 10.1042/BSR20200830 10.1042/BSR20200830 PMC7199454 32319516

[12] Guillén-Guío B, Acosta-Herrera M, Villar J, Flores C. Genetics of Acute Respiratory Distress Syndrome. eLS. 2016:1–9. 10.1002/9780470015902.a0026533

[13] Acosta-Herrera M, Lorenzo-Diaz F, Pino-Yanes M, Corrales A, Valladares F, Klassert TE (2015). Lung transcriptomics during protective ventilatory support in Sepsis-Induced Acute Lung Injury. PLoS ONE.

[14] Wang D, Li Y, Gu C, Liu M, Wang Y (2019). Identification of Key Pathways and genes of Acute Respiratory Distress Syndrome Specific Neutrophil phenotype. Biomed Res Int.

[15] Zheng F, Pan Y, Yang Y, Zeng C, Fang X, Shu Q (2022). Novel biomarkers for acute respiratory distress syndrome: genetics, epigenetics and transcriptomics. Biomark Med.

[16] Hawrylycz M, Miller JA, Menon V, Feng D, Dolbeare T, Guillozet-Bongaarts AL (2015). Canonical genetic signatures of the adult human brain. Nat Neurosci.

[17] Miller JA, Oldham MC, Geschwind DH (2008). A systems level analysis of transcriptional changes in Alzheimer’s disease and normal aging. J Neurosci.

[18] Dolinay T, Kim YS, Howrylak J, Hunninghake GM, An CH, Fredenburgh L (2012). Inflammasome-regulated cytokines are critical mediators of acute lung injury. Am J Respir Crit Care Med.

[19] Kangelaris KN, Prakash A, Liu KD, Aouizerat B, Woodruff PG, Erle DJ (2015). Increased expression of neutrophil-related genes in patients with early sepsis-induced ARDS. Am J Physiol Lung Cell Mol Physiol.

[20] Shi W, Oshlack A, Smyth GK (2010). Optimizing the noise versus bias trade-off for Illumina whole genome expression BeadChips. Nucleic Acids Res.

[21] Oldham MC, Langfelder P, Horvath S (2012). Network methods for describing sample relationships in genomic datasets: application to Huntington’s disease. BMC Syst Biol.

[22] Ritchie ME, Phipson B, Wu D, Hu Y, Law CW, Shi W (2015). Limma powers differential expression analyses for RNA-sequencing and microarray studies. Nucleic Acids Res.

[23] Yoav B, Daniel Y (2001). The control of the false discovery rate in multiple testing under dependency. The Annals of Statistics.

[24] Subramanian A, Tamayo P, Mootha VK, Mukherjee S, Ebert BL, Gillette MA (2005). Gene set enrichment analysis: a knowledge-based approach for interpreting genome-wide expression profiles. Proc Natl Acad Sci U S A.

[25] Liao Y, Wang J, Jaehnig EJ, Shi Z, Zhang B (2019). WebGestalt 2019: gene set analysis toolkit with revamped UIs and APIs. Nucleic Acids Res.

[26] Langfelder P, Horvath S (2008). WGCNA: an R package for weighted correlation network analysis. BMC Bioinformatics.

[27] Zhang B, Horvath S (2005). A general framework for weighted gene co-expression network analysis. Stat Appl Genet Mol Biol.

[28] Chin CH, Chen SH, Wu HH, Ho CW, Ko MT, Lin CY (2014). cytoHubba: identifying hub objects and sub-networks from complex interactome. BMC Syst Biol.

[29] Janky R, Verfaillie A, Imrichova H, Van de Sande B, Standaert L, Christiaens V (2014). iRegulon: from a gene list to a gene regulatory network using large motif and track collections. PLoS Comput Biol.

[30] Saguil A, Fargo MV (2020). Acute respiratory distress syndrome: diagnosis and management. Am Fam Physician.

[31] De Freitas Caires N, Gaudet A, Portier L, Tsicopoulos A, Mathieu D, Lassalle P (2018). Endocan, sepsis, pneumonia, and acute respiratory distress syndrome. Crit Care.

[32] Daurat A, Millet I, Roustan JP, Maury C, Taourel P, Jaber S (2016). Thoracic trauma severity score on admission allows to determine the risk of delayed ARDS in trauma patients with pulmonary contusion. Injury.

[33] Rubenfeld GD, Caldwell E, Peabody E, Weaver J, Martin DP, Neff M (2005). Incidence and outcomes of acute lung injury. N Engl J Med.

[34] Aulakh GK (2018). Neutrophils in the lung: “the first responders. Cell Tissue Res.

[35] Yang SC, Chen PJ, Chang SH, Weng YT, Chang FR, Chang KY (2018). Luteolin attenuates neutrophilic oxidative stress and inflammatory arthritis by inhibiting Raf1 activity. Biochem Pharmacol.

[36] Nguyen GT, Green ER, Mecsas J (2017). Neutrophils to the ROScue: mechanisms of NADPH oxidase activation and bacterial resistance. Front Cell Infect Microbiol.

[37] Teixeira G, Szyndralewiez C, Molango S, Carnesecchi S, Heitz F, Wiesel P (2017). Therapeutic potential of NADPH oxidase 1/4 inhibitors. Br J Pharmacol.

[38] Bime C, Zhou T, Wang T, Slepian MJ, Garcia JG, Hecker L (2016). Reactive oxygen species-associated molecular signature predicts survival in patients with sepsis. Pulm Circ.

[39] Galani V, Tatsaki E, Bai M, Kitsoulis P, Lekka M, Nakos G (2010). The role of apoptosis in the pathophysiology of Acute Respiratory Distress Syndrome (ARDS): an up-to-date cell-specific review. Pathol Res Pract.

[40] Kramer K, Harrington EO, Lu Q, Bellas R, Newton J, Sheahan KL (2003). Isoprenylcysteine carboxyl methyltransferase activity modulates endothelial cell apoptosis. Mol Biol Cell.

[41] Rounds S, Yee WL, Dawicki DD, Harrington E, Parks N, Cutaia MV (1998). Mechanism of extracellular ATP- and adenosine-induced apoptosis of cultured pulmonary artery endothelial cells. Am J Physiol.

[42] Broug-Holub E, Toews GB, van Iwaarden JF, Strieter RM, Kunkel SL, Paine R (1997). Alveolar macrophages are required for protective pulmonary defenses in murine Klebsiella pneumonia: elimination of alveolar macrophages increases neutrophil recruitment but decreases bacterial clearance and survival. Infect Immun.

[43] Johnston LK, Rims CR, Gill SE, McGuire JK, Manicone AM (2012). Pulmonary macrophage subpopulations in the induction and resolution of acute lung injury. Am J Respir Cell Mol Biol.

[44] Lomas-Neira J, Chung CS, Perl M, Gregory S, Biffl W, Ayala A (2006). Role of alveolar macrophage and migrating neutrophils in hemorrhage-induced priming for ALI subsequent to septic challenge. Am J Physiol Lung Cell Mol Physiol.

[45] Machado-Aranda D, Yu MVS, Dolgachev B, Hemmila V, Raghavendran MR (2014). Alveolar macrophage depletion increases the severity of acute inflammation following nonlethal unilateral lung contusion in mice. J Trauma Acute Care Surg.

[46] Narasaraju T, Yang E, Samy RP, Ng HH, Poh WP, Liew AA (2011). Excessive neutrophils and neutrophil extracellular traps contribute to acute lung injury of influenza pneumonitis. Am J Pathol.

[47] Durbin JE, Hackenmiller R, Simon MC, Levy DE (1996). Targeted disruption of the mouse Stat1 gene results in compromised innate immunity to viral disease. Cell.

[48] Meraz MA, White JM, Sheehan KC, Bach EA, Rodig SJ, Dighe AS (1996). Targeted disruption of the Stat1 gene in mice reveals unexpected physiologic specificity in the JAK-STAT signaling pathway. Cell.

[49] Minutti CM, Garcia-Fojeda B, Saenz A, de Las Casas-Engel M, Guillamat-Prats R, de Lorenzo A (2016). Surfactant protein A prevents IFN-gamma/IFN-gamma receptor Interaction and attenuates classical activation of human alveolar macrophages. J Immunol.

[50] Langlais D, Barreiro LB, Gros P (2016). The macrophage IRF8/IRF1 regulome is required for protection against infections and is associated with chronic inflammation. J Exp Med.

[51] Abelin JG, Harjanto D, Malloy M, Suri P, Colson T, Goulding SP (2019). Defining HLA-II ligand Processing and binding rules with Mass Spectrometry enhances Cancer Epitope Prediction. Immunity.

[52] Shams H, Klucar P, Weis SE, Lalvani A, Moonan PK, Safi H (2004). Characterization of a Mycobacterium tuberculosis peptide that is recognized by human CD4 + and CD8 + T cells in the context of multiple HLA alleles. J Immunol.

[53] Kruzel ML, Harari Y, Chen CY, Castro GA (2000). Lactoferrin protects gut mucosal integrity during endotoxemia induced by lipopolysaccharide in mice. Inflammation.

[54] Ochoa TJ, Chea-Woo E, Baiocchi N, Pecho I, Campos M, Prada A (2013). Randomized double-blind controlled trial of bovine lactoferrin for prevention of diarrhea in children. J Pediatr.

[55] Ochoa TJ, Pezo A, Cruz K, Chea-Woo E, Cleary TG (2012). Clinical studies of lactoferrin in children. Biochem Cell Biol.

[56] Hendrixson DR, Qiu J, Shewry SC, Fink DL, Petty S, Baker EN (2003). Human milk lactoferrin is a serine protease that cleaves Haemophilus surface proteins at arginine-rich sites. Mol Microbiol.

[57] Kruzel ML, Zimecki M, Actor JK (2017). Lactoferrin in a context of Inflammation-Induced Pathology. Front Immunol.

[58] Knox JJ, Cosma GL, Betts MR, McLane LM (2014). Characterization of T-bet and eomes in peripheral human immune cells. Front Immunol.

[59] Gruarin P, Maglie S, De Simone M, Haringer B, Vasco C, Ranzani V (2019). Eomesodermin controls a unique differentiation program in human IL-10 and IFN-gamma coproducing regulatory T cells. Eur J Immunol.

[60] Zhang P, Lee JS, Gartlan KH, Schuster IS, Comerford I, Varelias A, et al. Eomesodermin promotes the development of type 1 regulatory T (TR1) cells. Sci Immunol. 2017;2(10). 10.1126/sciimmunol.aah7152 10.1126/sciimmunol.aah7152 PMC5714294 28738016

