# Supplementary material for: Network analysis identifies a gene biomarker panel for sepsis-induced acute respiratory distress syndrome
Source: BMC Med Genomics. 2023 Jul 13;16:165. doi: 10.1186/s12920-023-01595-8 (PMC10339646; doi:10.1186/s12920-023-01595-8)
Supplement: Supplementary file 5 — Supplementary Material 5 [file 12920_2023_1595_MOESM5_ESM.docx]

# Supplementary figures


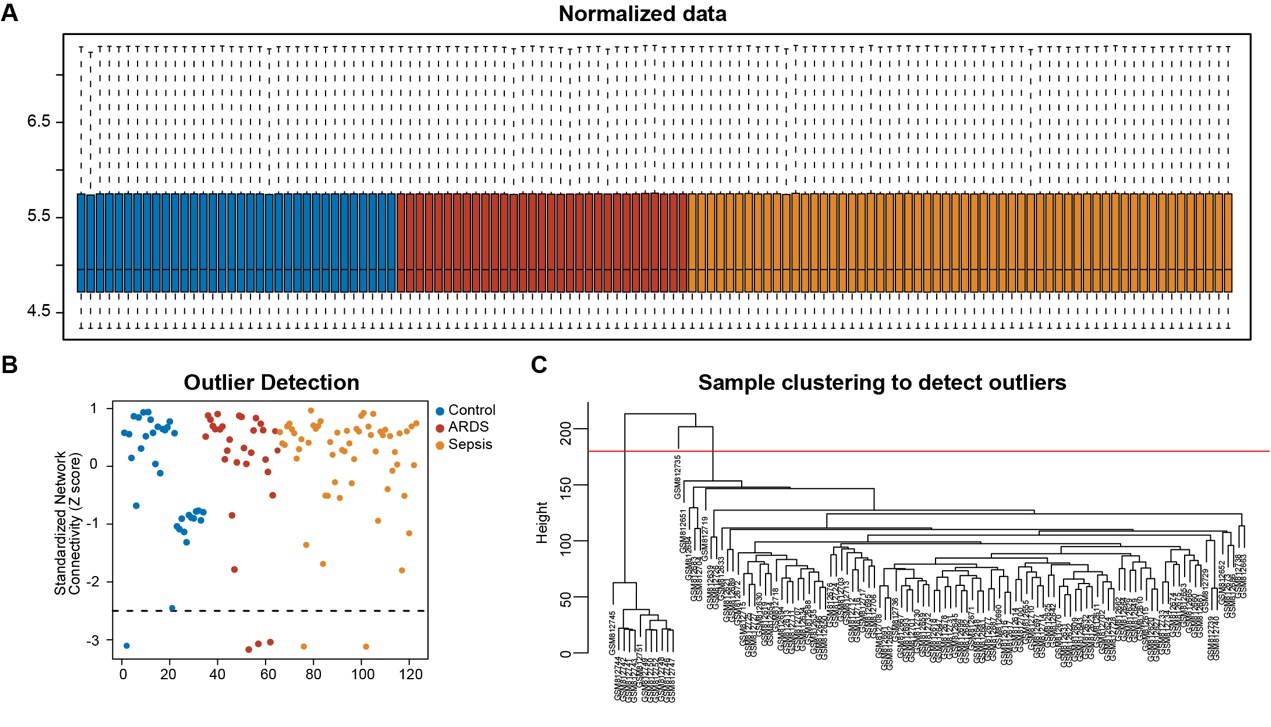


**Fig. S1. Data normalization.** **(A)** Boxplot of microarray data after normalization. **(B-C)** Identification of outliers by sample network method and cluster method.


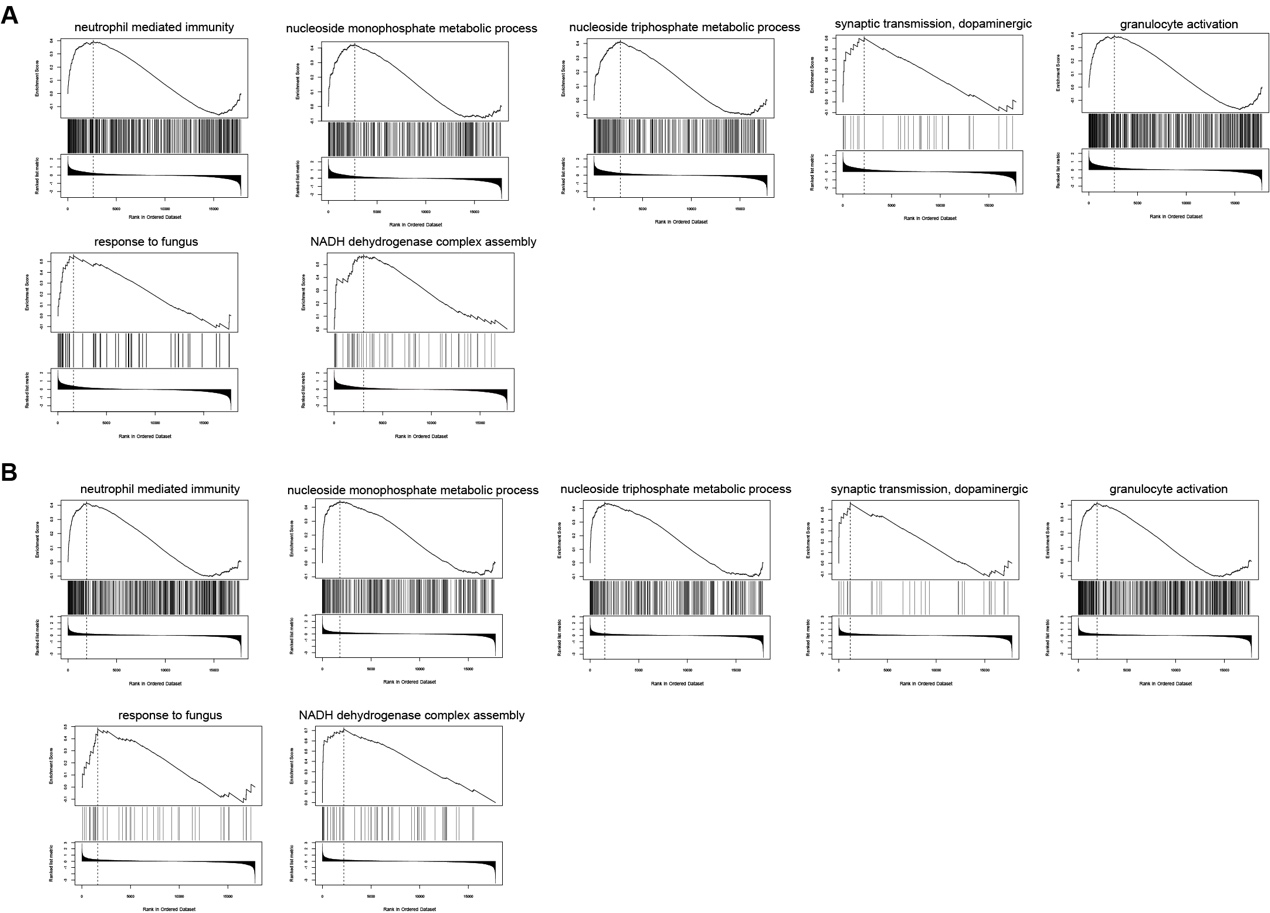


**Fig. S2. The 7 shared GO functions between sepsis-developed ARDS group and sepsis-alone group based on GSEA. (A)** Sepsis-developed ARDS group. **(B)** Sepsis-alone group. These functions were significantly positively enriched in both two groups.


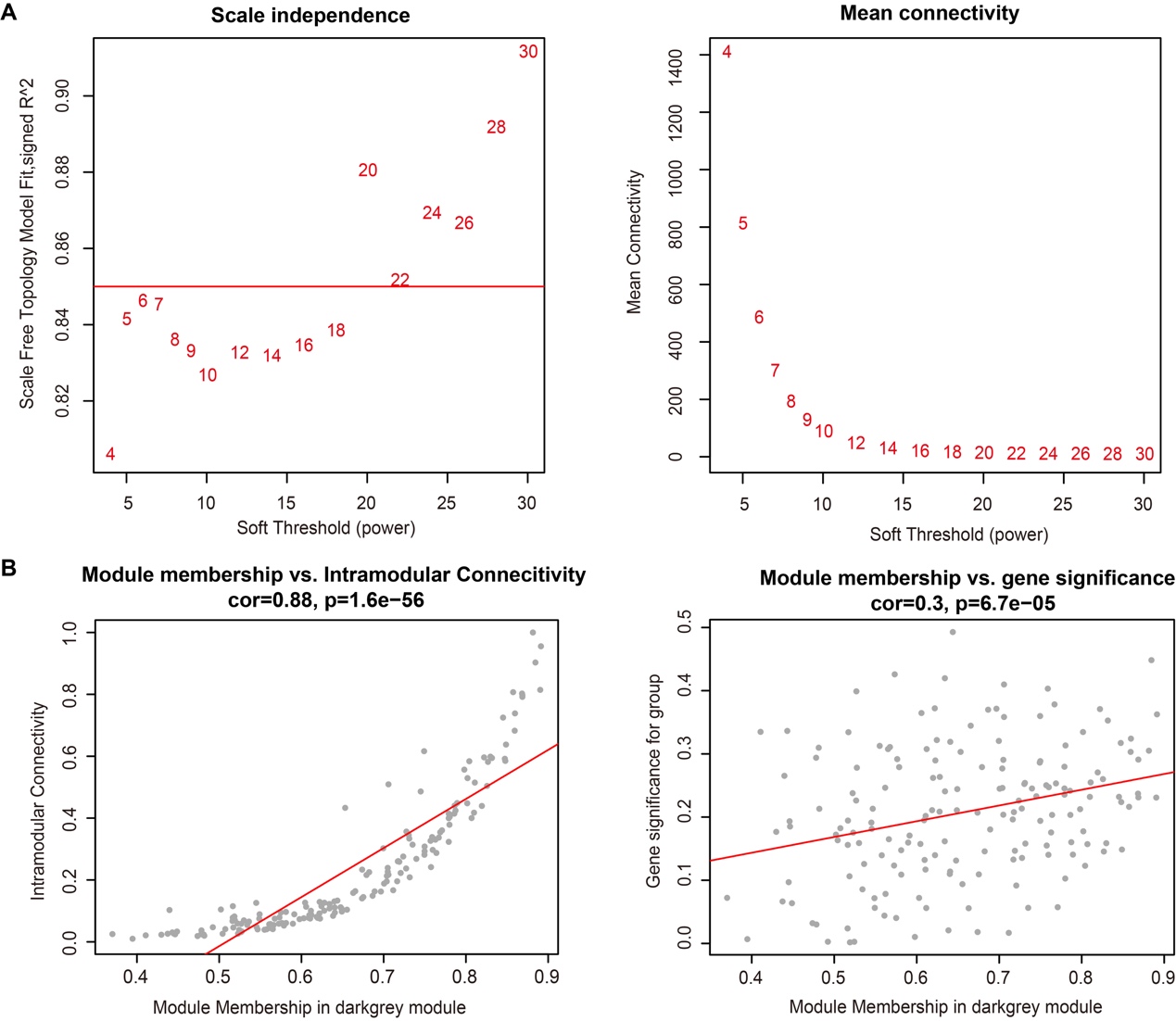


**Fig. S3. Power estimation for the construction of gene network. (A)** Soft-threshold procedure estimated 20 as the applicable power to fit the scale free network with the fit index greater than 0.85 and the average connectivity close to 0. **(B)** The module membership (MM) and intramodular connectivity (Kwithin) and gene significance (GS) of genes in the darkgrey module shows significant correlation (*p*<0.05).


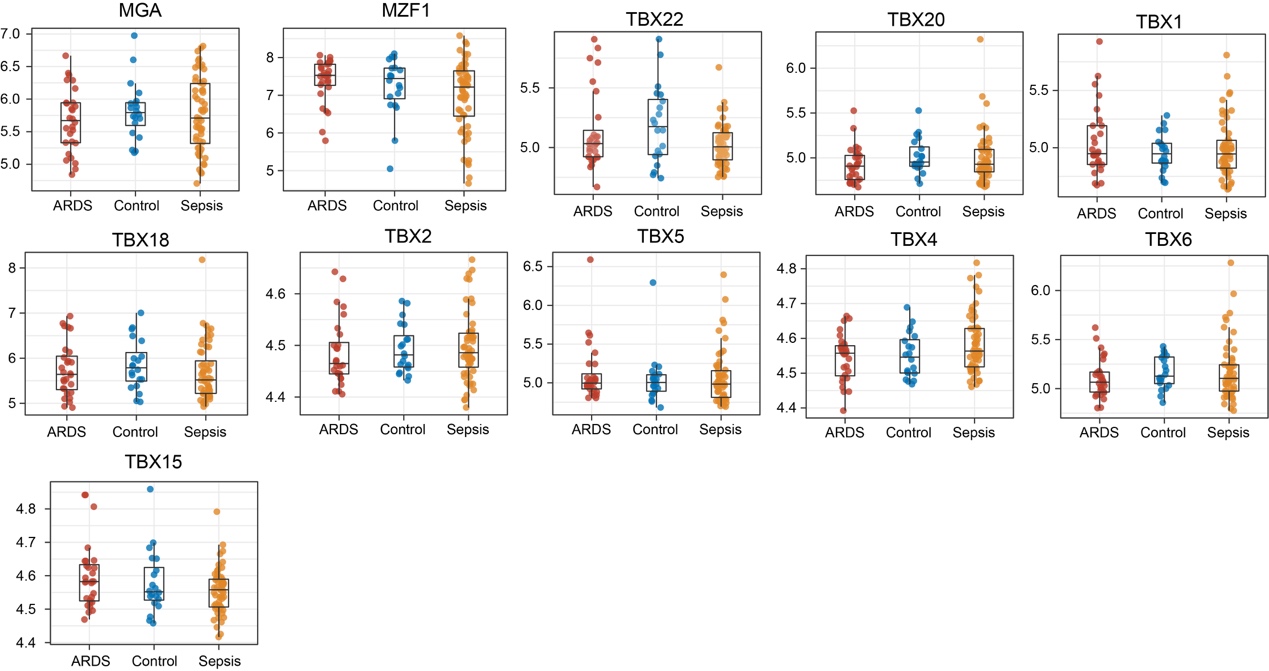


**Fig. S4. Expression of potential URFs.**

# Supplementary tables

**Table S1. Information of dataset.**

**Table S2. Differentially expressed genes and function analysis results based on GSEA and IPA software.**

**Table S3. Co-expressed gene module analysis.**

**Table S4. Degree of genes in PPI network and URFs prediction.**
